# Efficacy and safety of intradetrusor abobotulinumtoxinA and incobotulinumtoxinA in women with overactive bladder and the value of local anesthesia: a randomized clinical study

**DOI:** 10.1007/s00508-024-02412-7

**Published:** 2024-08-23

**Authors:** Niko Kavcic, Andrej Avsenak, Jan Zmazek, Tamara Serdinsek, Igor But

**Affiliations:** 1 Department of Urology, University Medical Centre Maribor, Maribor, Slovenia; 2 Faculty of Natural Sciences and Mathematics, University of Maribor, Maribor, Slovenia; 3 Division of Gynaecology, University Medical Centre Maribor, Maribor, Slovenia

**Keywords:** Botulinum toxin, Pain, Quality of life, Urinary incontinence

## Abstract

**Background:**

A non-inferiority clinical study evaluated the efficacy and safety of abobotulinumtoxinA vs. incobotulinumtoxinA intradetrusor injections in women with overactive bladder and urge urinary incontinence. Also, the effect of local anesthesia on the pain level of the procedure was assessed.

**Methods:**

Patients were randomized to receive 20 intradetrusor injections of either 300 U abobotulinumtoxinA or 100 U incobotulinumtoxinA. They were further randomized to receive either local anesthesia (40 ml 1% lidocaine solution) or placebo before botulinum toxin injection. Before the procedure and 4 months after the procedure each patient reported urinary incontinence episodes, frequency, nocturia, completed the Urogenital Distress Inventory (UDI-6) score, Incontinence Impact Questionnaire (IIQ-7), and Incontinence Quality of Life (I-QOL) questionnaire. Each patient completed a patient satisfaction survey 4 months after the procedure. During the procedure, the patients graded the pain intensity of every injection on a visual analog scale (VAS). The total score of each questionnaire was considered.

**Results:**

A total of 54 patients with a mean age of 66 ± 13 (SD) years completed the study. Total scores of UDI‑6, IIQ‑7, I‑QOL, patient satisfaction, urinary incontinence episodes, frequency, nocturia and VAS questionnaires did not show differences between the abobotulinumtoxinA (*n* = 26) or incobotulinumtoxinA (*n* = 28) group. Urinary retention requiring catheterization was noted in five patients. The VAS and patient satisfaction questionnaire values did not show significant differences between the group receiving bladder instillation with lidocaine solution (*n* = 28) or the group receiving placebo (*n* = 26).

**Conclusion:**

In women with overactive bladder and urge urinary incontinence where conservative treatment failed, abobotulinumtoxinA vs. incobotulinumtoxinA intradetrusor injections showed comparable results regarding improved clinical outcome and patient satisfaction. Local anesthesia before the procedure did not reduce the pain level in comparison with the placebo.

## Introduction

Overactive bladder (OAB) syndrome is defined by the International Continence Society as urinary urgency, with or without urgency urinary incontinence (UUI), and usually accompanied by frequency and nocturia in the absence of urinary tract infection or other obvious pathology [1, 2]. The prevalence of OAB in Europe has been estimated to be 15.6% for men and 17.4% for women, with an overall prevalence of 16.6% [3]. The national overactive bladder evaluation (NOBLE) study investigators determined the national prevalence of OAB in the USA for women to be 16.9%, 9.3% for OAB without UUI and 7.6% for OAB with UUI [4].

The OAB is a chronic condition that can have debilitating effects on the quality of life (QoL), including depression, sleep disorders, anxiety, social withdrawal, and sexual life impairment [5–7].

Treatment of OAB includes lifestyle therapy and behavioral and physical therapies. Antimuscarinic agents and beta‑3 adrenergic agonists are the main pharmacological treatment options. Surgical treatment is available for refractory disease, including botulinum toxin (BoNT) injection [8]. Botulinum toxin‑A is associated with significant symptom improvement in patients with idiopathic OAB; however, there is a higher incidence of postoperative urinary retention and urinary infection compared to placebo. Low-dose botulinum toxin‑A seems to provide the optimal balance between the benefits and adverse events. Trigone inclusion seems to be comparably safe and as effective as trigone-sparing injection [9]. A possible explanation for an advantage of trigone inclusion is the higher density of sensory fibers in the trigone area, consequently targeting the sensory complaints of OAB patients and patients with interstitial cystitis and/or bladder pain syndrome [10]. The evidence is slightly in favor of trigone inclusion [11].

Four different formulations of botulinum toxin, three BoNT‑A and one BoNT‑B, are commercially available in Europe and the USA: onabotulinumtoxinA (Botox®, Allergan Inc., Irvine, CA, USA), abobotulinumtoxinA (Dysport®, Ipsen Limited, Paris, France), iIncobotulinumtoxinA (Xeomin®, Merz Pharmaceuticals, Raleigh, NC, USA) and rimabotulinumtoxinB (Neurobloc/Myobloc®, Solstice Neurosciences Inc., San Francisco, CA, USA). Adequate clinical data are available for both onabotulinumtoxinA and abobotulinumtoxinA as a treatment option for neurogenic detrusor overactivity (NDO) [12]. To date, onabotulinumtoxinA and incobotulinumtoxinA have been investigated in several randomized, double-blind, head-to-head trials in patients with neurogenic diseases and no difference regarding clinical efficacy and safety profile was found [13, 14]. OnabotulinumtoxinA 100 U is licensed in Europe for treatment of OAB with persistent or refractory UUI in adults of both sexes [15, 16]; however, no prospective comparative studies have yet been performed on abobotulinumtoxinA and incobotulinumtoxinA in the urological field. The aim of this study was to compare the efficacy and safety of abobotulinumtoxinA and incobotulinumtoxinA in the treatment of refractory OAB with UUI. Moreover, we also aimed to evaluate the effect of lidocaine instillation on the pain level during the procedure.

## Material and methods

This was a prospective, randomized, single-blinded clinical study conducted at University Medical Centre Maribor between August 2018 and November 2022. National Medical Ethics Committee approval was obtained (approval number 0120-44/2018/5; date of approval 22 February2018). The study was retrospectively registered at ClinicalTrials.gov (ClinicalTrials.gov Identifier: NCT06250543, date of approval: 8.2.2024).

The primary aim of the study was to compare the efficacy of abobotulinumtoxinA and incobotulinumtoxinA treatment in women with OAB by evaluating the UDI‑6, IIQ‑7, and I‑QOL pretreatment and posttreatment scores.

The secondary aim of the study was to evaluate the effect of lidocaine instillation on the pain level during the procedure by evaluating the VAS score for patient satisfaction with the treatment, and to compare the occurrence of side effects between groups (urinary retention, urinary tract infections, UTI).

We included female patients with refractory OAB in whom previous treatment was unsuccessful and were planned for therapy with BoNT injection. The inclusion criteria were the following: female, age between 18 and 90 years, presence of urinary urgency with urgency urinary incontinence, residual urine below 150 ml, previous nonpharmacological conservative treatment inefficiency, anticholinergic or beta‑3 agonist treatment inefficiency.

The exclusion criteria were previous treatment with BoNT, pregnancy or breastfeeding, stress urinary incontinence (SUI), and any diseases or functional abnormalities that might affect bladder function (bladder stones, bladder tumors, bladder diverticula, demyelinating disease, Parkinson’s disease, spinal lesion, cerebral infarction, etc.). All patients signed the informed consent form prior to inclusion in the study.

Before treatment, each patient underwent a medical history and physical examination. Before BoNT injection, each patient completed three standardized questionnaires: Urogenital Distress Inventory (IU-6), Incontinence Impact Questionnaire (IIQ-7), and Incontinence Quality of Life (I-QOL). Each patient reported urinary incontinence episodes, frequency and nocturia before and 4 months after treatment. To extensively quantify the influence of UI, it is necessary to measure the level of symptoms of an individual and the magnitude to which they compromise their life. In urogynecology the utilization of questionnaires to measure outcomes has been increasing. The UDI‑6 and IIQ‑7 are well-known broadly used condition-specific questionnaires [17]. Urine dipstick test was routinely performed to exclude urinary tract infection (UTI). For all of the patients with UTI, treatment was rescheduled, the UTI was treated and another urine dipstick test was carried out. Patients were randomly assigned to one of two study arms by computer using an allocation ratio of 1:1. After BoNT treatment, the patients were invited to a follow-up visit after 1–2 weeks for systemic side effects evaluation, physical examination, measurement of residual urine, and urine dipstick test to diagnose and treat UTI. After 4 months, patients were seen to evaluate the treatment result and complete the IIQ‑7, UDI‑6, patient satisfaction, and I‑QOL questionnaires. Side effects were monitored for 5 months after the procedure.

The UDI‑6 is a questionnaire that comprises 6 items: “1—Frequent urination, 2—Leakage related to feeling of urgency, 3—Leakage related to activity, 4—Coughing or sneezing small amounts of leakage (drops), 5—Difficulty emptying the bladder, and 6—Pain or discomfort in the lower abdominal or genital area.” The scores range from 0 to 100. The higher the scores, the higher the disability of the patient [18]. Scores higher than 33.33 indicate higher distress caused by UI symptoms [17].

The IIQ‑7 is a questionnaire that comprises 7 items: “1—Household chores, 2—Physical recreation, 3—Entertainment activities, 4—Travel > 30 min away from home, 5—Social activities, 6—Emotional health (nervousness, depression, etc.), 7—Feeling frustrated.” The higher the scores, the higher the disability of the patient. The total score ranges from 0 to 100 [19]. Women who scored 9 or more on the IIQ‑7 questionnaire felt an impaired quality of life [17].

The I‑QOL is a questionnaire that evaluates the influence of urinary incontinence on health-related QOL. It comprises 22 items divided into 3 domains: an 8‑item domain evaluating the physical impact, a 9-item domain evaluating the psychological impact, and a 5-item domain evaluating the social impact. For all 22 items a total score is calculated, also for each domain scores are calculated. The scores range from 0 to 100. The higher the scores, the better the health status of the patient [5, 20].

Patient satisfaction was assessed using a 5-point scale: 1—very dissatisfied, 2—partially satisfied, 3—moderately satisfied, 4—very satisfied, and 5—exceptionally satisfied.

### Description of the intervention

The procedure was performed in an outpatient clinic. For antibiotic prophylaxis nitrofurantoin 100 mg twice daily per os was given on the day of the procedure. Patients were randomized to receive either 300 units of abobotulinumtoxinA (Dysport®) or 100 units of incobotulinumtoxinA (Xeomin®). They were further randomized to receive instillation anesthesia or placebo. The bladder was instilled with either 40 ml 1% lidocaine solution using a 16 Fr urethral Foley catheter or 40 ml 0.9% NaCl solution 30 min before the procedure. All patients received urethral lubrication gel containing lidocaine. Using rigid cystoscopy, 20 ml of normal saline was used to dilute each vial. A total of 20 evenly distributed intradetrusor injections were administered. Each site was injected with 1 ml and 2 injections included the trigone area. During the procedure, the patients scored the pain intensity of every injection on a visual analog scale (VAS). All procedures were performed by one experienced surgeon.

### Sample size calculation

For this non-inferiority trial, a group sample size of 64 subjects was required to reach 80% power of the test with a significance level (alpha) of 0.05. The clinically important non-inferiority limit for the UDI‑6 questionnaire was set at 18. The expected difference between the means of both treatments was assumed to be 0 [17, 21]. The sample size was calculated by using Sealed Envelope Ltd. 2012. Power calculator for continuous outcome non-inferiority trial.

### Statistical analysis

Statistical analysis was performed using SPSS (Version 29.0, IBM, Armonk, New York, United States). Basic patient characteristics were calculated using elemental descriptive statistics. Non-parametric statistical tests were used to compare data between groups. Continuous data from independent samples were analyzed using the Mann-Whitney U test, whereas comparisons of continuous data within matched samples employed the Wilcoxon signed-rank test. In all analyses, a two-sided *p*-value < 0.05 was considered significant. Considering the multiple hypotheses being tested in the study (a total of 10), the Bonferroni correction was applied to adjust for multiple comparisons.

## Results

**Fig. 1:**
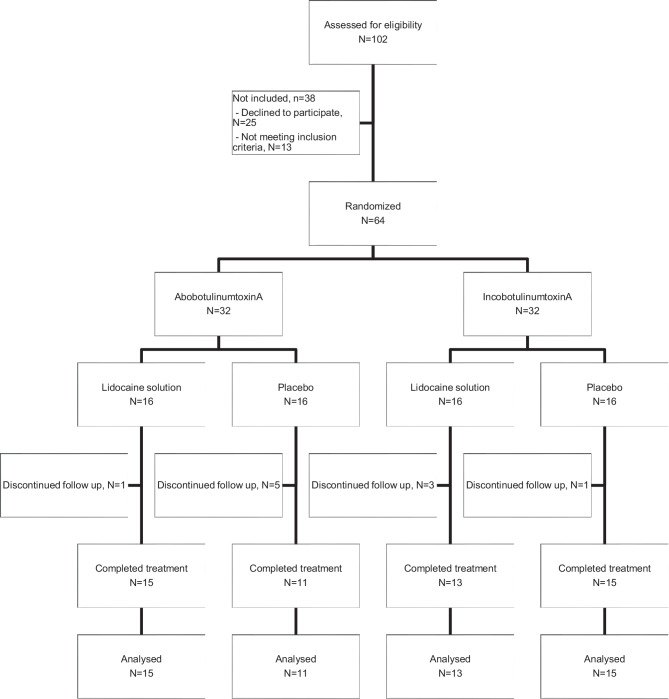
Flow chart of patient enrolment and the follow-up process

In the study period 102 patients were invited to participate in the study and assessed for eligibility. Of these, 25/102 (24.5%) declined to participate, and 13/102 (12.7%) did not meet the inclusion criteria. A total of 64/102 (62.7%) patients entered the study and were randomized into the abobotulinumtoxinA group—group 1 (32 patients) or incobotulinumtoxinA group—group 2 (32 patients). All patients received the intervention; however, 10/64 (15.6%) were lost during follow-up. In the end, 54/64 (84.3%) patients completed the study, with 26 being treated with abobotulinumtoxinA and 28 with incobotulinumtoxinA (Fig. 1). Moreover, 28 patients received bladder instillation with lidocaine solution and 26 patients received placebo (Fig. 1).

Both groups were comparable regarding age and baseline urinary incontinence episodes, frequency, nocturia, UDI‑6, IIQ‑7, and I‑QOL results (Table 1).

**Table 1:** Patient baseline characteristics for both groups (age, pretreatment questionnaire scores, pretreatment incontinence episodes, frequency and nocturia)

Variable	Group 1 *N* = 26	Group 2 *N* = 28	*p*-value
Age [years, mean ± SD, (range)]	65.3 ± 12.6 (41–82)	66.4 ± 12.7 (39–86)	0.782
Pretreatment UDI‑6 [mean ± SD, (range)]	66.1 ± 18.9 (22–100)	58.4 ± 21.5 (14–100)	0.187
Pretreatment IIQ‑7 [mean ± SD, (range)]	58 ± 22.9 (5–100)	59.1 ± 25.9 (5–100)	0.748
Pretreatment I‑QOL [mean ± SD, (range)]	46.3 ± 16.6 (23.6–88.2)	43.4 ± 16.3 (20.0–77.3)	0.456
Pretreatment incontinence episodes	4.88 ± 3.18 (1–12)	6.32 ± 4.74 (1–18)	0.417
Pretreatment frequency	9.85 ± 3.63 (4–16)	9.71 ± 2.99 (5–15)	1.000
Pretreatment nocturia	2.19 ± 1.65 (0–7)	2.79 ± 1.66 (0–7)	0.116

*SD* standard deviation

After treatment, a significant improvement in all questionnaire scores was observed in both groups (Table 2). Also, a significant improvement in urinary incontinence episodes and frequency was observed. Only nocturia in group 1 did not improve significantly.

**Table 2:** Comparison of questionnaire scores, urinary incontinence episodes, frequency and nocturia within groups

Group	Questionnaire	Pretreatment [mean ± SD, (range)]	Posttreatment [mean ± SD, (range)]	*p*-value
Group 1 *N* = 26	UDI‑6	66.1 ± 18.9 (22–100)	52.0 ± 26.1 (11–100)	0.003**
	IIQ‑7	58 ± 22.9 (5–100)	40.2 ± 28 (0–100)	0.010*
	I‑QOL	46.3 ± 16.6 (23.6–88.2)	59.3 ± 23.5 (28.2–97.3)	0.008*
	Incontinence episodes	4.88 ± 3.18 (1–12)	2.12 ± 3.08 (0–12)	< 0.001*
	Frequency	9.85 ± 3.63 (4–16)	7.77 ± 2.32 (4–14)	0.003
	Nocturia	2.19 ± 1.65 (0–7)	1.81 ± 2.37 (0–11)	0.191
Group 2 *N* = 28	UDI‑6	58.4 ± 21.5 (14–100)	37.9 ± 27.7 (6–100)	< 0.001*
	IIQ‑7	59.1 ± 25.9 (5–100)	26.5 ± 27.1 (0–100)	< 0.001*
	I‑QOL	43.4 ± 16.3 (20.0–77.3)	69.4 ± 23.4 (20.0–99.1)	< 0.001*
	Incontinence episodes	6.32 ± 4.74 (1–18)	2.18 ± 3.12 (0–13)	< 0.001*
	Frequency	9.71 ± 2.99 (5–15)	7.64 ± 7.50 (4–15)	< 0.001
	Nocturia	2.79 ± 1.66 (0–7)	1.79 ± 1.42 (0–5)	0.008

*SD* standard deviation, *statistically significant difference

**Fig. 2:**
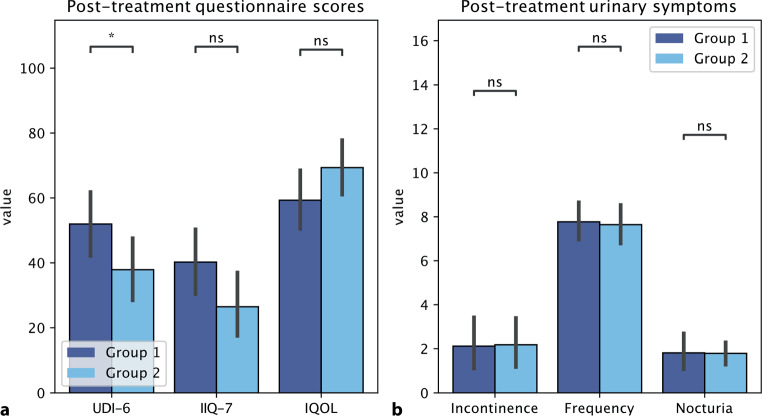
**a** Comparison of post-treatment questionnaire scores between groups. **b** Comparison of post-treatment urinary incontinence episodes, frequency and nocturia. *statistically significant difference, *ns* not significant

However, when comparing questionnaire scores and urinary incontinence episodes, frequency and nocturia between groups after treatment, there was only a statistically significant difference in UDI‑6 scores in favor of group 2 (*p*-value 0.042) (Fig. 2).

The mean VAS score during the procedure in patients with instillation anesthesia was 4.3 ± 1.7 (range 2–9), compared to 4.6 ± 1.5 (range 1–7) in patients in the placebo group. This difference did not reach statistical significance (*p*-value = 0.236).

The mean patient satisfaction score was 3.5 ± 1.2. The difference in the satisfaction score for both BoNT types did not reach statistical significance (*p*-value = 0.172). The difference in the satisfaction score for anesthesia type did not achieve statistical significance (*p*-value = 0.748).

Urinary retention requiring catheterization was noted in 5/54 (9.3%) patients, 4 in group 1, and 1 in group 2 (*p*-value = 0.159). UTI requiring antibiotic treatment developed in 16/54 (29.6%) patients, 10 in group 1, and 6 in group 2 (*p*-value = 0.133).

## Discussion

The injection of BoNT is a highly effective treatment for refractory OAB. Four different formulations of botulinum toxin are available; however, clinical data on efficacy in neurogenic and non-neurogenic OAB treatment are mostly available for onabotulinumtoxinA although other subtypes of BoNT‑A are also commonly used, including abobotulinumtoxinA and incontobotulinumtoxinA, and data suggest that a dose of 100 U onabotulinumtoxinA offers the best efficacy/tolerability ratio in non-neurogenic OAB patients [11, 13, 14]. As stated by the recent review article by Michel et al., studies with abobotulinumtoxinA in OAB are scarce [11]. A small prospective study randomized women with idiopathic OAB to receive either 300 U or 500 U of abobotulinumtoxinA trigone-sparing injections and concluded that intravesical abobotulinumtoxinA injections at 500 U improve symptoms and quality of life for a longer period of time than 300 U, despite the fact that one woman required transient clean intermittent catheterization [11, 22]. Ravindra et al. compared onabotulinumtoxinA and abobotulinumtoxinA in non-neurogenic OAB patients. They prospectively included 207 patients, 101 in the onabotulinumtoxinA cohort and 106 in the abobotulinumtoxinA cohort. They observed similar reductions in daytime frequency, nocturia, and incontinence episodes after the treatment, with no difference in duration of effect [23]. A small trial comparing 500 U of abobotulinumtoxinA injections including the trigone to the group not including the trigone, in idiopathic OAB patients, described a greater treatment effect on OAB symptoms in the trigone-including group [24].

Only a few studies are available on the efficacy of incobotulinumtoxinA in patients with OAB. Asafu-Adjei et al. and Grishin et al. established that incontobotulinumtoxinA is effective in patients with detrusor overactivity and OAB [25, 26]. Grishin et al. used 200 U of incontobotulinumtoxinA in idiopathic OAB patients [26]. Giannantoni et al. discovered that incontobotulinumtoxinA is not inferior to onabotulinumtoxinA in patients with neurogenic detrusor overactivity [14]. No other study has yet compared the efficacy of abobotulinumtoxinA and incobotulinumtoxinA. Different formulations of botulinum toxin are not bioidentical and have different potency [27]. A conversion factor between onabotulinumtoxinA and abobotulinumtoxinA of 1:2.5 was suggested by Grosse et al. [28]; however, this assumption was not scientifically proven. It is believed a variable conversion rate of the two toxins between 1:2 and 1:3 is applicable [29]. Observations from the neurologic field indicate that incobotulinumtoxinA was as effective as onabotulinumtoxinA with a comparable safety profile when a clinical conversion ratio of 1:1 or 1:1.2 was used [30]. Based on these observations, we decided to use abobotulinumtoxinA and incobotulinumtoxinA in a ratio of 3:1.

In summary, the overall study rationale of our research was to compare incobotulinumtoxinA sporadically studied in the idiopathic OAB setting and abobotulinumtoxinA which has been studied in the therapy of idiopathic OAB before; however, both formulations of BoNT have been extensively studied and compared with onabotulinumtoxinA in treating patients with neurogenic detrusor overactivity.

We discovered that both abobotulinumtoxinA and incobotulinumtoxinA significantly improve patients’ symptoms as evaluated by standardized questionnaires, and there were no statistically significant differences in primary outcomes between groups.

In the study by Lange et al. [31], only 7% of medical practitioners used two different types of formulations of BoNT, while the rest only used onabotulinumtoxinA. A possible hindrance, in their opinion, might be the fact that, apart from onabotulinumtoxinA, the use of other BoNT products for OAB is off-label in all three countries where the survey was conducted. They further argued that because of the possibility of decreased efficacy of certain formulations in the case of neutralizing antibodies, it might be required to use a different formulation. Hence, it is advisable that medical practitioners are used to the application of different products [31, 32]. IncobotulinumtoxinA was approved by the FDA in 2011 for the treatment of cervical dystonia and blepharospasm. The rationale behind its potential use in OAB treatment is that incobotulinumtoxinA is a highly purified form of BoNT with no complexing proteins and could potentially be less immunogenic in patients. According to some authors, this may be particularly advantageous in patients with neurogenic disease, who often receive injections in multiple organs [25].

The adverse events profile of intravesical BoNT‑A injections in idiopathic OAB patients is dominated by an increase in post void residual urine volume, sometimes even urinary retention, the need for clean intermittent catheterization and UTI [11], hence we focused on the follow-up of these entities. In our study, the rate of urinary retention requiring catheterization was low (9.3%) compared to other studies in this field, and there were no statistically significant differences between groups. For example, Ravindra et al. reported a symptomatic urinary retention rate of 42% in idiopathic OAB patients receiving abobotulinumtoxinA and 23% in patients receiving onabotulinumtoxinA [23]. In general, the rate of urinary retention after BoNT treatment is highly variable. For onabotulinumtoxinA, it ranges between 5.4% and 43%. The difference in the reported rates is largely dependent on the definition of urinary retention, patient population, and the units of onabotulinumtoxinA used [33]. UTIs requiring treatment developed in 29.6% of patients, which is comparable to results reported by other authors. Mohamed-Ahmed et al. found that 21% of their patients developed UTIs after the first BoNT treatment, and 23% of patients developed recurrent UTIs [34].

BoNT application can be performed with the patient under local or general anesthesia. Sedation-free intradetrusor botulinum toxin‑A injection using intravesical lidocaine and flexible endoscopy is a well-tolerated and safe procedure to perform in an office setting [35]. An online survey of members of the Canadian Urological Association showed that of those performing treatment using local anesthetics, most (66%) used the instillation of a lidocaine solution for analgesia [36]. The results of a multinational online survey of urogynecologists in Germany, Austria, and Switzerland showed that for BoNT use in OAB, half of all urogynecologists performed the procedure using general anesthesia, while local anesthesia was utilized by 39% of participants. While board-certified urogynecologists and high-volume surgeons used local anesthesia significantly more often, general obstetricians and gynecologists more often used spinal anesthesia or no anesthesia at all [31]. No study was found comparing any type of anesthesia to a placebo for BoNT, and it is therefore unknown if local anesthesia is superior to a placebo [31]. A randomized controlled trial found that alkalinized lidocaine solution was not superior to lidocaine gel for pain control during intravesical BoNT injections [37]. In a study by Schurch B et al. [38], EMDA (electromotive drug administration)-enhanced instillation of lidocaine enabled sufficient anesthesia of the bladder wall that ensured a painless application of the botulinum‑A toxin injections into the detrusor muscle. As the different formulations of botulinum toxin might affect the pain level of the procedure, it is worth noting that our results show no difference in the VAS between both drug groups. Interestingly, there was no difference between the pain level of the procedure and patient satisfaction in the group receiving lidocaine instillation and the placebo group.

The advantage of our study is the practical implication of using alternative forms of BoNT‑A in treating OAB. The research study addresses a previously underexplored area by comparing two BoNT‑A formulations that have received limited attention in research on the treatment of women with OAB and UUI. This provides additional data to the current knowledge base. We have also questioned the routine use of local anesthesia for pain reduction. As a result, future medical practice could change in omitting lidocaine instillations and discover more effective forms of local anesthesia.

The disadvantage of our study is the relatively high dropout rate of our patients (10 out of 64), partially due to the SARS-COV‑2 pandemic. Hence, the required sample size of 64 patients was not reached. Larger, randomized, controlled studies investigating the non-inferiority of either abobotulinumtoxinA or incobotulinumtoxinA and the non-inferiority of placebo in comparison to lidocaine use are required.

OAB is a prevalent chronic health condition with a very negative impact on QoL [3, 5]. All approved therapies for OAB are just improving the symptoms and have to be used for longer time periods, which is problematic if the efficacy does not meet the expectations of the patient, hence the adherence to therapy is low [11, 39]. Increasing the repertoire of botulinum toxin formulations and other novel therapeutic options, together with the identification of different subsets of OAB patients with various biomarkers, will allow more efficient treatment tailored to each subgroup of patients [11].

## Conclusion

This prospective clinical study demonstrated that intradetrusor injections of 300 U abobotulinumtoxinA vs. 100 U incobotulinumtoxinA showed non-inferior clinical outcomes in women with OAB and UUI who failed conservative treatment. Additionally, the pain level of the procedure was not affected by the use of local anesthesia before the procedure.
